# Predicting the possibility of African horse sickness (AHS) introduction into China using spatial risk analysis and habitat connectivity of *Culicoides*

**DOI:** 10.1038/s41598-022-07512-w

**Published:** 2022-03-10

**Authors:** Shan Gao, Zan Zeng, HaoNing Wang, FangYuan Chen, LiYa Huang, XiaoLong Wang

**Affiliations:** 1 College of Wildlife & Protected Area, Ministry of Education, Northeast Forestry University, 26 Hexing Road, Xiangfang District, Harbin, 150040 Heilongjiang Province People’s Republic of China; 2 Key Laboratory of Wildlife Diseases and Biosecurity Management of Heilongjiang Province, 26 Hexing Road, Xiangfang District, Harbin, 150040 Heilongjiang Province People’s Republic of China; 3 Department of Vascular Surgery, Changhai Hospital, Second Military Medical University, Shanghai, 200433 People’s Republic of China; 4 School of Geography and Tourism, Harbin University, Harbin, 150086 Heilongjiang Province People’s Republic of China; 5 The Second Geomatics Cartography Institute, Ministry of Natural Resource, Harbin, 150086 Heilongjiang Province People’s Republic of China; 6 Changbai Mountain Academy of Sciences, Antu, 133613 Jilin Province People’s Republic of China

**Keywords:** Ecology, Diseases

## Abstract

African horse sickness (AHS) is a devastating equine infectious disease. On 17 March 2020, it first appeared in Thailand and threatened all the South-East Asia equine industry security. Therefore, it is imperative to carry out risk warnings of the AHS in China. The maximum entropy algorithm was used to model AHS and *Culicoides* separately by using climate and non-climate variables. The least cost path (LCP) method was used to analyze the habitat connectivity of *Culicoides* with the reclassified land cover and altitude as cost factors. The models showed the mean area under the curve as 0.918 and 0.964 for AHS and *Culicoides*. The prediction result map shows that there is a high risk area in the southern part of China while the habitats of the *Culicoides* are connected to each other. Therefore, the risk of introducing AHS into China is high and control of the border area should be strengthened immediately.

## Introduction

The African Horse Sickness (AHS) is a non-contagious, arboviral and highly infectious disease^1^. AHS is caused by the African Horse Sickness Virus (AHSV), which belongs to the genus of Orbivirus in the family of Reoviridae^2^. The clinical signs of AHS are usually classified into four forms, which usually cause respiratory and circulatory damage^3^. AHSV infects mainly equids with high mortality rates up to 90% in horses. The mortality of mules infected with AHSV was about 50%, and that of donkeys was about 10%. Zebra is the natural storage host of AHSV and does not show symptoms after infection^4,5^. Because of its high mortality and strong transmission ability, AHS has a high negative economic impact in the countries where the disease occurs and seriously affects the international trade of horses^6^. The World Organization for Animal Health (OIE) has listed AHSV as a notifiable disease, and it is the only equine disease for OIE to observe official recognition status^7^.

AHSV is transmitted by hematophagous biting midges of the genus *Culicoides* (Diptera: Ceratopogonidae)^8^. A proven vector is *Culicoides imicola*, while other *Culicoides spp*., notably C. bolitinos may also play a role in transmission^9,10^. At present, nine serotypes (AHSV-1 to AHSV-9) have been identified, while serotype 9 (AHSV-9) is the most widely distributed one^11,12^. AHSV is prevalent in tropical and subtropical regions of Africa, south of Sahara, West from Senegal to Ethiopia and Somalia in the East, and extends to South Africa^13^. Outside Africa, outbreaks of the virus has caused devastating losses in indigenous horses in the Middle East, India, Pakistan, North Africa and Europe^14^. In February 2020, Thailand reported a case of AHS for the first time in Southeast Asia and OIE World Assembly of Delegates Resolution that the “AHS free country” status of Thailand is suspended with effect from 27 March 2020^15^. subsequently, the “AHS free country” status of Malaysia is suspended with effect from August 6, 2020. With regard to the current situation of AHS, some of these recent outbreaks occurred in provinces of Thailand that share borders with Myanmar, Cambodia, Malaysia and Laos. If they are not brought under control, AHS will directly threaten the equine industry in Southeast Asia and its neighboring countries. Although, the spread of AHS threatens neighboring countries, if Thailand’s neighboring countries use currently licensed vaccines, it will lead to loss of the AHS freedom status, resulting in losses to the economy and trade^16^. Therefore, early surveillance of the outbreak is very important. It is being reported that Cambodia and China are already testing horses for AHS^17^.

China is identified as a non-epidemic area of AHS by OIE^2^. China shares a 4061 km border with Myanmar, Laos, and Vietnam in the Southwest, where transportation is more convenient by many ports and channels. The increasing global trade and the climate changes may facilitate the spread of vector-borne diseases, as shown by recent outbreaks of Bluetongue and Smallenberg viruses and demonstrating the rising viral transmission by *Culicoides* in non-endemic areas^18,19^. There are a large number of entry-exit population and goods there, which are vulnerable to the epidemic. And Thailand and southwest China have a hot and humid climates. Multiple hosts and insect vectors have been found there^20^. The wide geographic distribution of *Culicoides* spp. and potential year-round activity poses an inherent risk of AHSV spreading among neighboring countries^21^.

In recent years, the frequent occurrence of emerging infectious diseases has brought serious harm to social and economic development. Early warning can greatly reduce the losses caused by the late pandemic. As the straight-line distance between Thailand and Yunnan province, China is only 110 km and shares a very similar situation in biodiversity, climate, landscape, and population of both human and livestock, if the *Culicoides spp* of habitat connecting perfect and will not hinder the spreading of AHS naturally. Therefore, the aim of this study is to use the maximum entropy model (MaxEnt) to identify high risk areas for AHS occurrence in Southwest China and its neighboring countries and the connectivity of landscapes among the research area of *Culicoides* using the LCP model, thereby revealing the possibility of introducing AHS into China.

## Methods

### Research area

**Figure 1:**
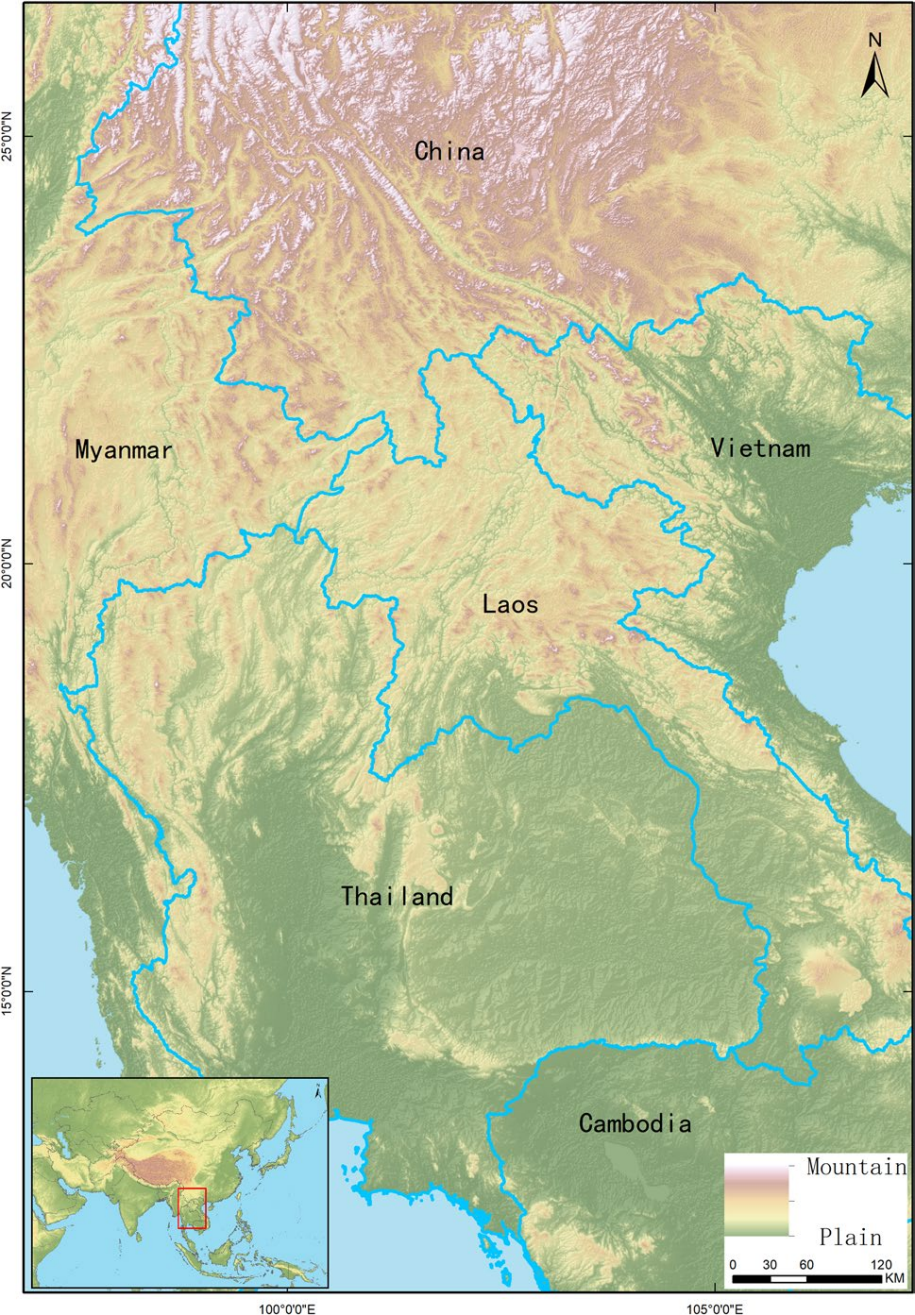
Research area. The elevation depicted by the digital elevation model (DEM). DEM was obtained from USGS Earth Explorer (https://www.earthexplorer.usgs.gov); the boundary was obtained from Natural Earth (http://www.naturalearthdata.com/), which is a schematic line illustrating the relative position of each country and should not be re-used or misinterpreted for any political reason.

Our research area (Fig. 1) is located within 18.75–37.03^◦^N, 69.84–99.51^◦^E, including Yunnan and part of (Guangxi, Sichuan, Guizhou) provinces in China, Myanmar, Laos, Vietnam and Thailand. The landscape within the research area is complex and changeable, with an elevation high in the north and low in the south. The tropical and subtropical monsoon climate is expressed as high temperature, heavy rainfall, and high humidity. There are a high diversity of *Culicoides* species and other vectors, which support the transmission of various arboviruses^22^.

### Data collection and processing

The Culicoides present points (n = 95) were obtained by screening the Web of Science, Scopus, Science Direct, PubMed and Chinese National Knowledge Infrastructure (CNKI) from 2000 to 2021 years. For some Culicoides records that record the locations of discovery or capture, we use Google Maps to convert them into coordinates. The outbreak locations of AHS (n = 18) were collected from the OIE reports from 2020.02 to 2021.06 years. Five fundamental environmental predictor categories relevant for modeling, namely climate, terrain, vegetation, livestock distribution and human impact, were used (Table 1).

**Table 1:** Data layer and source, raster/vector, value range/categories and specification of the unit of measurement/impact (proxy) included in the models.

	Source	Variable value range or categories (type)	factors included in the Culicoides models	factors included in the AHS models
Climate^a^	CHELSA	Current 1979–2013/Forecast 2041–2060	Y	Y
Monthly P	Ibid	0 to 545 / 0 to 581 mm/month	Y	Y
Monthly mean T	Ibid	−17.5 to 29 / −15.5 to 31.3 °C	Y	Y
Monthly min T	Ibid	−25.3 to 25.2 / −23.4 to 27.7 °C	Y	Y
Monthly max T	Ibid	−11.9 to 32.6 ℃/ −9.3 to 34.9 °C	Y	Y
Bioclimatic (19)	Ibid	Supplementary data Table A1	Y	Y
**Terrain**	ASTER-GDEM ^b^			
DEM	Ibid	−553 to 7845 m a.s.l	Y	Y
**Human impact**				
Population	WorldPop ^c^	0.3 to 3940.9 persons/km^2^	Y	Y
**Categorial**				
Land cover/Vegetation	ESA^d^	Categorical	Y	Y
**Livestock distribution data** ^e^				
Spatial distribution for cattle	GWL3	0–344,862 individual/km2	Y	N
Spatial distribution for sheep	GWL3	0–344,862 individual/km2	Y	N
Spatial distribution for goats	GWL3	0–344,862 individual/km2	Y	N
Spatial distribution for buffaloes	GWL3	0–344,862 individual/km2	Y	N
Spatial distribution for horses	GWL3	0–344,862 individual/km2	Y	Y
Spatial distribution for pigs	GWL3	0–344,862 individual/km2	Y	N
Spatial distribution for chickens	GWL3	0–344,862 individual/km2	Y	N
Spatial distribution for ducks	GWL3	0–344,862 individual/km2	Y	N

^a^ T = temperature; P = precipitation. Source: http://chelsa-climate.org/.

^b^ Source: http://www.gscloud.cn/.

^c^ Source: https://www.worldpop.org/.

^d^ Land cover: Cropland, Herbaceous, Tree, Shrubland, Grassland, Urban areas, Bare areas, Water bodies and Permanent snow and ice.

^e^Source: http://www.fao.org/livestock-systems/.

### Spatial models of AHS and *Culicoides*

All spatial data were preprocessed by standard operations in ArcGIS 10.2 and projected in UTM-WGS-1984^23^. Where necessary, we resampled to 30 arc-seconds. We use the stepped minimum distance (0, 5, 10, 15, 20…35, 40 km) to filter the recorded points of AHS and *Culicoides* to reduce the spatial autocorrelation used the SDM Toolbox v1.1c in ArcGIS 10.2, and use them to build the pre-model. Then, according to the performance of the model (AUC value), the most suitable filtering distance is estimated^24^. The multicollinearity reduction for climate predictors and non-climate predictors was done as follows, respectively. First, we used a Principal Component Analysis (PCA) to select major predictors^25^. Next, MaxEnt model analysis was carried out to eliminate the factors with a low contribution rate^26^ and eliminate predictor variables with a high standard deviation (SD) based on visual observation of the response curves^27^. Finally, we performed Variance Inflation Factor (VIF) analysis, which < 10 indicates low multicollinearity^28^. The final predict variables and the filtered points were input into the MaxEnt model to construct the *Culicoides* (model I) and AHS (model II). The results of the two models are a fuzzy overlay to obtain a prediction map.

### Analysis of habitat

#### Connectivity of *Culicoides* by least cost path

**Figure 2:**
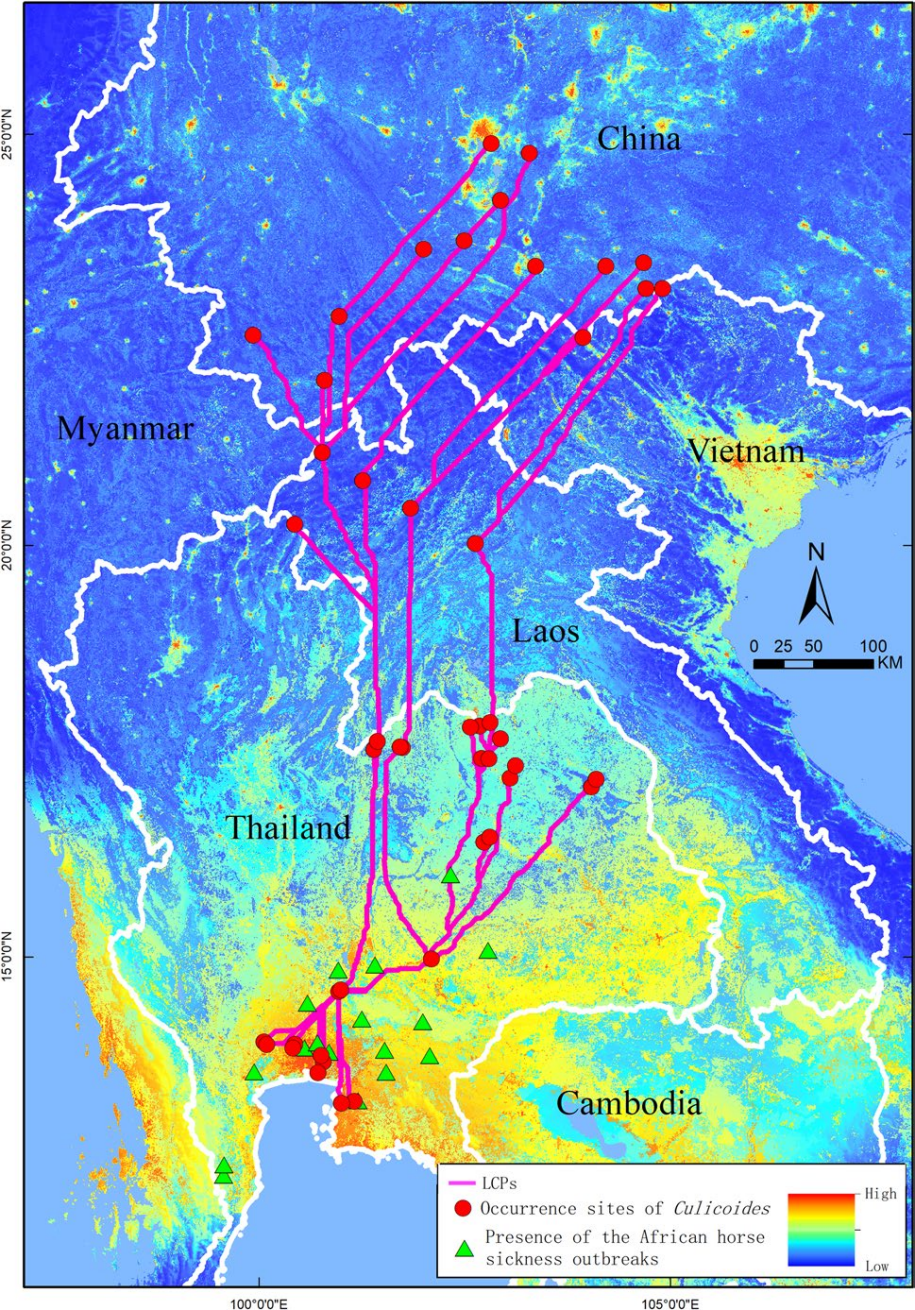
AHS and *Culicoides* overlying high-risk areas and transboundary Least Cost Paths for *Culicoides*. This map was made in ArcGIS 10.6 using the resulting rasters produced by MaxEnt. Red circle means occurrence sites of *Culicoides* and green triangle means African horse sickness outbreaks in the research area.occurrence sites of Culicoide. The pink lines shows the LCPs between of *Culicoides*. The boundary was obtained from Natural Earth (http://www.naturalearthdata.com/), which is a schematic line illustrating the relative position of each country and should not be re-used or misinterpreted for any political reason.

The least cost (LCP) is the shortest path moving between two individuals and can be used to analyze connectivity in landscapes^29^. We created a cost surface for *Culicoides* dispersal using reclassified land cover and elevation as cost factors. Two factors were reclassified following the Jenks natural breaks method^30^. The resistance value is set to 1 and 9, and the higher the value, the higher the resistance. That is, when the value is 1, the resistance is the smallest and the *Culicoides* is the easiest to pass; when the value is 9, the resistance is the largest and the *Culicoides* is the most difficult to pass. We determine the cost values of the land cover and elevation according to *Culicoides* movement preference. Factors such as elevations between −883 to 4200 m, forest, shrubland, Mosaic herbaceous, Grassland were assigned with the lowest cost value (= 1). Raster cells with elevations > 4200 m, bare areas, Permanent snow, and ice, etc. were assigned the highest cost value (= 9). The *Culicoides* presence points in the study area are clustered by ArcGIS, and the LCP is constructed between clusters of points (Fig. 2).

## Results

**Figure 3:**
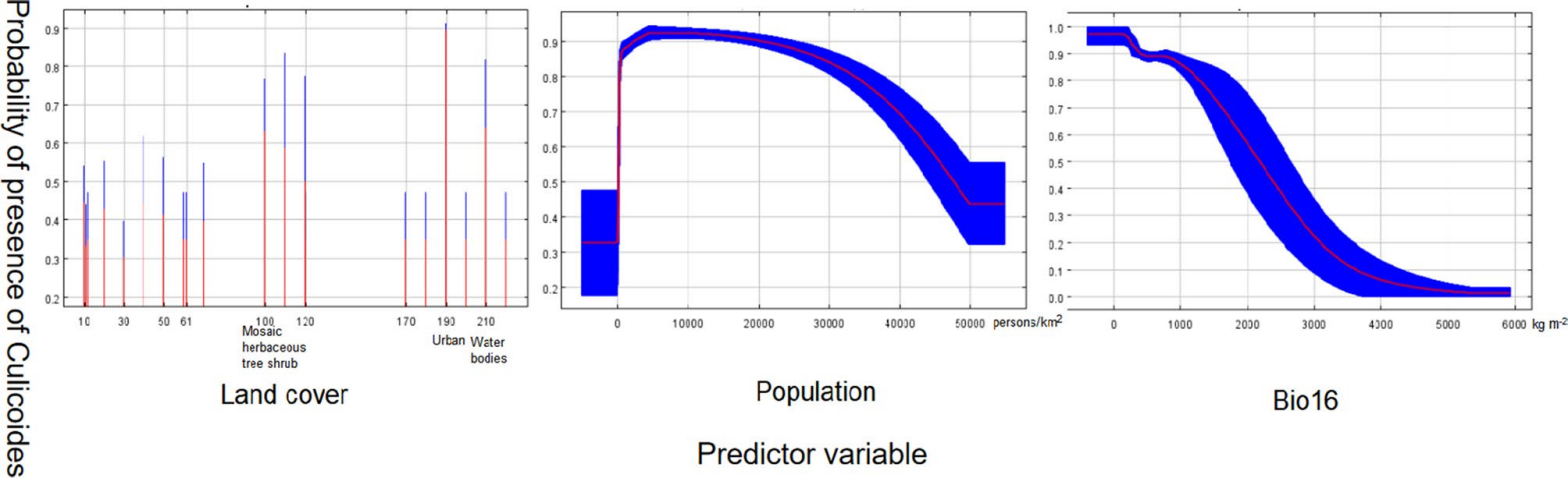
The response curves of Model I. The curves show the mean response (red) and the mean standard deviation (blue).

Model I: 85 *Culicoides* presence points remained after filtering by 20 km. After the multicollinearity reduction, the mean monthly precipitation amount of the wettest quarter (bio16), population and land cover are saved. The VIF among predictors is 1.014–1.742, which was in line with the low multicollinearity standard (< 10). Further, the AUC value (0.918) and the standard deviation (SD) (0.021) identified a robust model. The response curve of the predictor is shown in Fig. 3, and the relative contributions of predictors are shown in Table 2.

**Table 2:** Estimates of contributions of important predictor variables to the model.

Model I (*Culicoides*)			Model II (AHS)		
Variable	Contribution %	Permutation importance	Variable	Contribution %	Permutation importance
Landcover	55.8	23.9	Bio7	54	60.6
Population	40.4	61.6	Land cover	26	10.9
Bio16	3.8	14.6	Prec12	20	28.5

**Figure 4:**
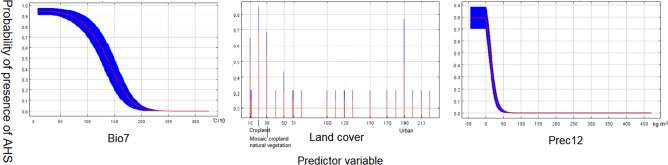
The response curves of Model II. The curves show the mean response (red) and the mean standard deviation (blue).

Model II: 17 AHS outbreak points remained after filtering by 10 km. After multicollinearity reduction, the annual range of air temperature (bio7), precipitation amount of December (prec12) and land cover are saved. The VIF among predictors is 1.006–1.062. The AUC value (0.964) and the standard deviation(SD)(0.006) are identical to a robust model. The response curve of the predictor is shown in Fig. 4, and the relative contributions of predictors are shown in Table 2.

The probability of risk of AHS is shown in Fig. 2. The high and middle risk areas are mainly distributed in Thailand, northern and Western Vietnam, northern Laos, Yunnan, Guangxi and Sichuan Province of China. The *Culicoides* presence points are clustered in four groups. There were multiple possible paths among the four clusters connecting all the habitats of *Culicoides* (Fig. 2).

## Discussion

AHS is a truly devastating disease from both an animal welfare perspective and in respect of the economic damage that it causes^31^. The European experience of bluetongue in small ruminants, which has become endemic throughout the region, and that blue tongue and African horse fever virus are both transmitted by the same insect vectors, reminds us that AHS is a potential threat throughout Southeast Asia^32^. Although China is currently a country without an AHS epidemic, the outbreaks of AHS in Thailand and Malaysia warn that the African horse plague has begun to spread in Southeast Asia^33^. Although China has no direct border with Thailand, the distance between China's western border and Thailand's border is only 110 km, the nearest outbreak point of AHS is only 600 km away from the border of China. The density of horse population on the southern border of China is the highest in China, which is far more than that in Thailand and neighboring countries. Therefore, we must do a good job in disease warnings. Once AHS enters China, it will cause immeasurable consequences to China^34,35^.

The surveillance and control of common disease-transmitted vectors, such as *Culicoides*, is of great importance for the prevention and control of vector-borne infectious diseases^36^. According to the World Health Organization (WHO) recommendation, one of the most effective strategies for eliminating vector-borne infectious diseases is to control the vectors or intermediates host of the pathogens. At present, significant progress has been made in the study of *Culicoides* spp in China, but there is a lack of in-depth research on the biological vector *Culicoides*. The geographical distribution, ecological habits, seasonal fluctuation and population advantage of several *Culicoides* which can transmit animal-borne disease virus in China have not been studied thoroughly and comprehensively. Some species of *Culicoides* are also distributed in China, and some of them are closely related to the same subgenus as the main vectors of disease. Especially in the southern border areas of China, it is very difficult to conduct a systematic and comprehensive survey and monitoring of *Culicoides*. It is difficult to capture *Culicoides* in the southwest border areas of China because of the complex terrain, more forest and shrub coverage. Therefore, it is not only beneficial for us to understand the suitable habitat of *Culicoides* but also to solve the vacancy in the survey and monitoring by using the MaxEnt method to analyze the suitable niche model.

So far, the vectors and potential hosts of AHS have not been fully understood^37^. Although there are many studies on AHS, the outbreak in Thailand is the first in Asia since 1961^38^. Therefore, the study of vectors of AHS is still limited^21^. Studies have shown that approximately 30 species of midges belonging to the genus *Culicoides* have been associated with AHSV transmission and replication, Not only *Culicoides* spp. play a role in transmission, other occasional modes of transmission are mosquitoes-Culex, Anopheles and Aedes spp.^39^. our study is only for the confirmed field transmission vector (*Culicoides spp*) of AHSV for Modeling and analysis. Although *Culicoides* species live in various habitats, they are mostly wet warm and enriched in organic matter of animal or plant origin^40^. The main factors affecting the distribution of *Culicoides* in our model are land cover, population density and (bio16). Cities and towns are important factors affecting the distribution of *Culicoides* in vegetation types. People and livestock in urban areas provide more food for *Culicoides*. Buildings and animal feeding circles in cities and towns provide more life for *Culicoides*, including wintering habitat. Previous studies have shown that *Culicoides* have a higher density of birds and livestock houses in many places and have a tendency to birds and livestock houses. Male midges feed on plant sap, and only female midges feed on blood^41^. Female midges have a wide range of blood-sucking, and their adults usually live in sheltered places such as trees, bamboo forests, weeds and caves. Therefore, Mosaic tree, shrub and herbaceous cover in land cover also show a high impact^42^. Population density also has a great impact on the distribution of *Culicoides*. Population density reflects the degree of urbanization and the ecological environment change caused by human activities^43^. Bio16 is the wettest season precipitation, although the survival of *Culicoides* needs more water, the lack of activity when it rains, especially in the breeding period, and too much precipitation will have a negative impact on the flight of *Culicoides* feeding, their flight may be limited, too much precipitation will also lead to a decrease in temperature, which is not conducive to the survival of *Culicoides*, and the response curve is consistent. The main influencing factors of AHS in our model are bio7, land cover, prec12. It has been studied that there is a close relationship between expression temperature and disease^44^. The temperature will affect the survival time of the virus in the environment and affect the activity and range of the host and transmission medium. If the temperature changes too much in that year, the incidence of disease will decrease. The vegetation type is dominated by farmland, and farmland also provides food for livestock, so the number of livestock population is large, while female *Culicoides* mainly rely on blood meal as a food source, so the possibility of AHS transmission may be greatly increased^45^. The average precipitation in December also had an impact on the disease. With the increase of precipitation in December, the incidence of the disease decreased sharply and then remained flat. Studies have shown that the incidence rate of vector-borne diseases will decrease with the increase of precipitation at low temperatures^46^.

Because *Culicoides* have the ability of long-distance transmission, we use the connectivity model to determine whether the *Culicoides* habitat is connected^34^. Within connectivity models, least cost paths(LCP), which are the shortest path between two points with maximum efficiency for a moving individual, have been advocated as an effective, operational, and flexible approach to analyzing connectivity in heterogeneous landscapes. It constructs a resistance surface based on the assumed "cost" of landscape components relative to species movement and determines the cumulative least cost path between points. This approach can integrate geographic and behavioral information and comprehensively calculate the available paths. According to previous studies, altitude and vegetation are the main resistance factors that affect the activity of *Culicoides*^47^, so we also built the resistance layer based on these two factors. The analysis of the epidemic situation in Thailand and Malaysia shows that AHS has the possibility of cross-border transmission in Southeast Asia. In the study area, the climate is characterized by high temperatures throughout the year, almost frost-free, average temperature between 0℃ and 15℃, and high forest coverage. Therefore, the environment is very suitable for the survival and development of *Culicoides*. LCP method also confirmed that the habitat of *Culicoides* is connected, and *Culicoides* is likely to move between suitable habitats to spread the disease of AHS. But The importation and cross-border movement of livestock carrying the virus could also contribute to the spread of the disease, and the effect of wind on the movement of *Culicoides* cannot be ignored, but cannot be control^48,49^.

China is a big horse breeding country, and the number of horses is at the forefront of the world. In 2019, 3.671 million horses were on hand in China, accounting for 6% of the world's total, ranking fifth. According to industry statistics, the horse industry's output value of the whole industry chain is about 70 billion yuan^50^. In southern China, there are a large number of horses and a large number and variety of AHS vectors. Once the AHS virus vectors enter China, it will cause a fatal blow to China's horse industry. We also consider the high mortality of AHS and the lack of transmission mechanism in wild equine. As most of the wild equine species in China are endangered, once AHS enters China, the wild equine species in China will face a great threat. If infected with AHS, the wild animal population may be extinct. Therefore, the prevention and control of the introduction of AHS are of great significance not only to the protection of the equine breeding industry in China but also to the prevention of the spread of wild endangered species. At present, there is no AHS in China, but ahs has the conditions for cross-border transmission. China should early warn the disease, strengthen the monitoring of insect vectors in border areas, and strengthen the supervision of border horse trade. Once the epidemic is found, timely preventive measures should be taken to prevent a huge impact on China's horse industry. This study reveals the impact of environmental variables on the risk of AHS and warns the risk and possibility of AHS introduction into China, so we should prevent and control AHS in advance. It is being reported that Cambodia and China are already testing horses for AHS.

## Supplementary Information

Supplementary Information.
