# Supplementary material for: Predicting the possibility of African horse sickness (AHS) introduction into China using spatial risk analysis and habitat connectivity of Culicoides
Source: Sci Rep. 2022 Mar 10;12:3910. doi: 10.1038/s41598-022-07512-w (PMC8913660; doi:10.1038/s41598-022-07512-w)
Supplement: Supplementary file 1 — Supplementary Information. [file 41598_2022_7512_MOESM1_ESM.docx]

**Title:** Predicting the possibility of African horse sickness (AHS) introduction into China using spatial risk analysis and habitat connectivity of *Culicoides*

**Authors**: Shan Gao^1, 2&^, Zan Zeng^1, 2&^, HaoNing Wang^3^, FangYuan Chen^4^, LiYa Huang^5^, XiaoLong Wang ^1, 2*^

## Supporting information

**Table 1** Permeability values used to classify the environmental maps according to *Culicoides* movement preference

| Layer | Influence (%) | Value/categories | Cost value^a^ | References |
| --- | --- | --- | --- | --- |
| Elevation | 50 | -833-4200 | 1 | (Duan, Bellis et al. 2019, Duan, Yang et al. 2021) |
|  |  | **≥**4200 | 9 |  |
| Land cover^b^ | 50 | Rainfed cropland | 9 | (Leta, Fetene et al. 2019) |
|  |  | Herbaceous cover | 1 |  |
|  |  | Irrigated or post-flooding | 1 |  |
|  |  | Mosaic cropland (>50%)/natural vegetation | 1 |  |
|  |  | Mosaic natural vegetation | 1 |  |
|  |  | Evergreen broadleaved | 1 |  |
|  |  | Deciduous broadleaved(>15%) | 1 |  |
|  |  | Deciduous broadleaved(>40%) | 1 |  |
|  |  | Evergreen needleleaved(>15%) | 1 |  |
|  |  | Evergreen needleleaved(>40%) | 1 |  |
|  |  | Evergreen needleleaved(15-40%) | 1 |  |
|  |  | Mosaic tree and shrub (>50%) / herbaceous cover (<50%) | 1 |  |
|  |  | Mosaic herbaceous cover (>50%) | 1 |  |
|  |  | Shrubland | 1 |  |
|  |  | Grassland | 1 |  |
|  |  | Sparse vegetation | 1 |  |
|  |  | Flooded tree cover(saline water) | 1 |  |
|  |  | Flooded tree cover(fresh/saline/brakish water) | 1 |  |
|  |  | Urban areas | 1 |  |
|  |  | Bare areas | 9 |  |
|  |  | Consolidated bare | 9 |  |
|  |  | Unconsolidated bare | 9 |  |
|  |  | Water bodies | Nodata |  |
|  |  | Permanent snow and ice | 9 |  |

^a^ 1 and 9 means the crossing cost of *Culicoides* is from low to high

^b^ Land cover has no frequency data, and scale value is defined according to the description in references

Duan, Y. L., et al. (2019). "Potential vectors of bluetongue virus in high altitude areas of Yunnan Province, China." **12**(1).

Duan, Y. L., et al. (2021). "Isolation of Tibet Orbivirus from Culicoides jacobsoni (Diptera, Ceratopogonidae) in China." **14**(1).

Leta, S., et al. (2019). "Modeling the global distribution of Culicoides imicola: an Ensemble approach." **9**(1).

**Table 2** Principal component analysis results of climate variables of African horse sickness.(The value is the load value of each variable, and its absolute value is directly proportional to its importance in the principal component)

| Component  Variable | 1 | 2 | 3 |
| --- | --- | --- | --- |
| bio1 | 0.993 |  |  |
| temp10 | 0.993 |  |  |
| tmax11 | 0.988 |  |  |
| temp9 | 0.985 |  |  |
| tmin5 | 0.985 |  |  |
| tmin9 | 0.984 |  |  |
| temp2 | 0.984 |  |  |
| tmin3 | 0.984 |  |  |
| tmin6 | 0.981 |  |  |
| tmin8 | 0.978 |  |  |
| bio8 | 0.977 |  |  |
| tmax1 | 0.976 |  |  |
| temp8 | 0.976 |  |  |
| tmin7 | 0.976 |  |  |
| tmax10 | 0.975 |  |  |
| tmax9 | 0.970 |  |  |
| tmin10 | 0.968 |  |  |
| temp3 | 0.965 |  |  |
| temp5 | 0.965 |  |  |
| tmax12 | 0.964 |  |  |
| bio10 | 0.958 |  |  |
| temp7 | 0.956 |  |  |
| temp6 | 0.956 |  |  |
| temp4 | 0.951 |  |  |
| tmax2 | 0.950 |  |  |
| temp11 | 0.948 |  |  |
| tmax8 | 0.942 |  |  |
| tmin2 | 0.939 |  |  |
| temp1 | 0.918 |  |  |
| bio11 | 0.906 |  |  |
| bio9 | 0.905 |  |  |
| tmax5 | 0.905 |  |  |
| tmax7 | 0.892 |  |  |
| tmax6 | 0.891 |  |  |
| temp12 | 0.884 |  |  |
| tmax3 | 0.878 |  |  |
| bio5 | 0.872 |  |  |
| tmax4 | 0.872 |  |  |
| tmin11 | 0.855 |  |  |
| bio17 |  | 0.934 |  |
| prec10 |  | 0.926 |  |
| prec1 |  | 0.924 |  |
| bio14 |  | 0.921 |  |
| prec12 |  | 0.921 |  |
| bio7 |  | -0.904 |  |
| bio2 |  | -0.900 |  |
| bio4 |  | -0.881 |  |
| bio12 |  |  | 0.978 |
| bio16 |  |  | 0.971 |
| prec5 |  |  | 0.953 |
| prec8 |  |  | 0.951 |
| prec7 |  |  | 0.944 |
| prec6 |  |  | 0.939 |
| prec9 |  |  | 0.911 |
| bio13 |  |  | 0.911 |

Extraction Method: Principal Component Analysis.

Rotation Method: Varimax with Kaiser Normalization.

1. Rotation converged in 6 iterations.

**Table 3** Principal component analysis results of climate variables of *Culicoides*

(The value is the load value of each variable, and its absolute value is directly proportional to its importance in the principal component)

| Component  Variable | 1 | 2 | 3 | 4 | 5 |
| --- | --- | --- | --- | --- | --- |
| bio4 | 0.973 |  |  |  |  |
| bio3 | 0.953 |  |  |  |  |
| bio7 | 0.946 |  |  |  |  |
| tmax1 | 0.934 |  |  |  |  |
| tmax2 | 0.930 |  |  |  |  |
| temp1 | 0.918 |  |  |  |  |
| tmax12 | 0.917 |  |  |  |  |
| temp2 | 0.913 |  |  |  |  |
| bio9 | 0.912 |  |  |  |  |
| bio11 | 0.912 |  |  |  |  |
| tmax3 | 0.907 |  |  |  |  |
| temp12 | 0.897 |  |  |  |  |
| temp3 | 0.897 |  |  |  |  |
| tmin1 | 0.889 |  |  |  |  |
| bio6 | 0.889 |  |  |  |  |
| tmin2 | 0.881 |  |  |  |  |
| tmin3 | 0.871 |  |  |  |  |
| tmax11 | 0.871 |  |  |  |  |
| tmin12 | 0.865 |  |  |  |  |
| tmax7 |  | 0.969 |  |  |  |
| tmax8 |  | 0.967 |  |  |  |
| temp7 |  | 0.944 |  |  |  |
| temp8 |  | 0.938 |  |  |  |
| bio10 |  | 0.906 |  |  |  |
| bio5 |  | 0.892 |  |  |  |
| tmin7 |  | 0.891 |  |  |  |
| bio8 |  | 0.891 |  |  |  |
| tmin8 |  | 0.888 |  |  |  |
| tmax6 |  | 0.873 |  |  |  |
| prec7 |  |  | 0.963 |  |  |
| prec8 |  |  | 0.918 |  |  |
| bio16 |  |  | 0.897 |  |  |
| bio13 |  |  | 0.874 |  |  |
| bio19 |  |  |  | 0.926 |  |
| prec12 |  |  |  | 0.894 |  |
| prec3 |  |  |  |  | 0.935 |

Extraction Method: Principal Component Analysis.

Rotation Method: Varimax with Kaiser Normalization.

1. Rotation converged in 7 iterations.


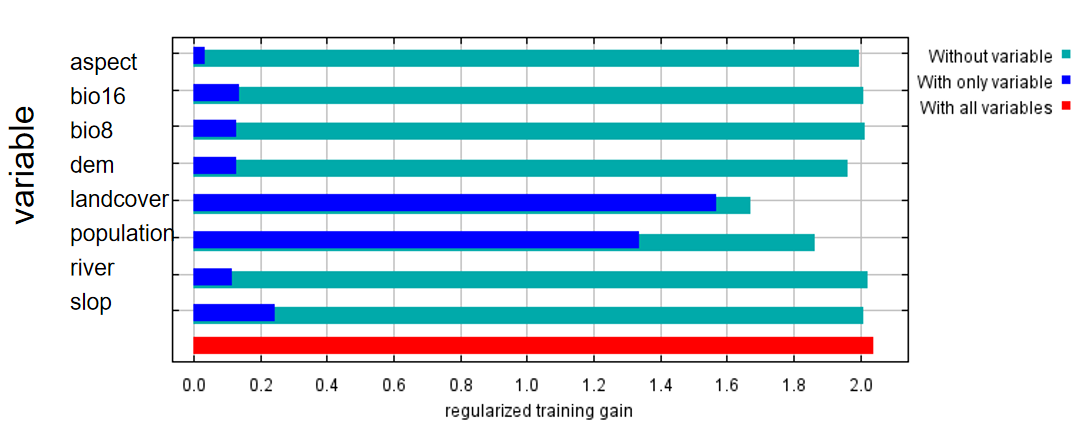


**Figure 1** Jackknife of regularized training gain for *Culicoides*


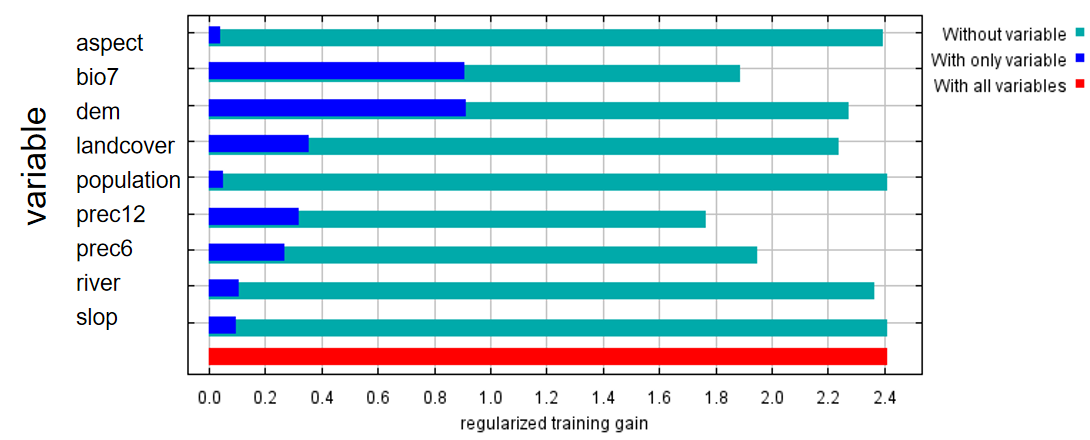


**Figure 2** Jackknife of regularized training gain for African horse sickness
